# Dispersion Inhibits Thermal Mitigation of *Pseudomonas aeruginosa* Biofilms on Self‐Heating Surfaces

**DOI:** 10.1002/mbo3.70283

**Published:** 2026-04-06

**Authors:** Parham Parnian, Paraskevi K. Zoga, Haydar A. S. Aljaafari, Hannah Chicchelly, Michael Toops, Eric Nuxoll

**Affiliations:** ^1^ Department of Chemical & Biochemical Engineering University of Iowa Iowa City Iowa USA; ^2^ Center for Biocatalysis and Bioprocessing University of Iowa Iowa City Iowa USA; ^3^ Department of Pharmaceutical Sciences and Experimental Therapeutics University of Iowa Iowa City Iowa USA; ^4^ Department of Chemical Engineering University of Technology Baghdad Iraq

**Keywords:** biofilm, hyperthermia, medical device infection, pseudomonas aeruginosa, thermoelectric device

## Abstract

Bacterial biofilms on medical implants are a major problem, typically requiring explantation and replacement of the biofilm‐colonized implant. Thermal mitigation of these biofilms *in situ* has shown great promise in the laboratory, where the thermal shock can be most precisely delivered by immersion in hot media. Clinical implementation requires delivering the shock from the implant surface, however, leaving the surroundings at a cooler temperature. This study hypothesized that bacteria may rapidly, reversibly disperse into the cooler surroundings to partially evade the shock and tested this hypothesis by thermally shocking *Pseudomonas aeruginosa* biofilms on thermoelectric devices under media with different heat sink conditions. The time scale and equilibrium constant of this dispersion were investigated in ambient temperature immersion studies, and the effect of thermal shock on bacterial dispersion rate was investigated in a flow cell using biofilms grown on thermoelectric devices. The results showed that biofilms equilibrate with surrounding media in seconds, that a small fraction of bacteria in the biofilm are much less prone to dispersion, that thermal shock triggers an immediate increase in dispersion, and that shocking biofilms via their substrate in cooler surrounding decreases shock efficacy compared to shocks where the surrounding's temperature approaches that of the substrate.

## Graphical abstract

Thermal mitigation of biofilm infections is promising in the lab. Clinical translation requires heat to be delivered from the implant surface while leaving surrounding tissue cooler. This study shows that bacteria may rapidly and reversibly disperse into these cooler areas, reducing treatment efficacy.

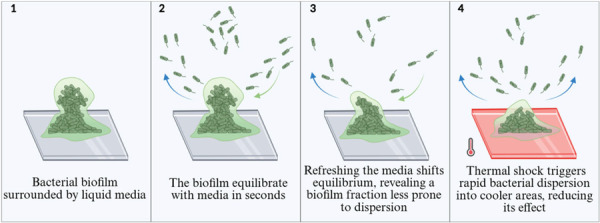

## 1 Introduction

Biofilms are large populations of single or multi‐species bacterial cells surrounded by a self‐synthesized extracellular polymeric substance (Costerton et al. 1999; Flemming et al. 2023; Ruhal and Kataria 2021). Being able to adhere to inert or living surfaces, biofilms are the principal cause of assorted chronic infections not only in internal organs but also on medical devices including prosthetics, pacemakers, vascular catheters, *etc (*Khardori and Yassien 1995; Lebeaux and Ghigo 2012; Lebeaux et al. 2014; Hosseini et al. 2019; Deshmukh‐Reeves et al. 2025). In general, 65‐80% of human infections (Lebeaux et al. 2013; Høiby et al. 2015; Yang et al. 2025), and 70% of nosocomial infections are attributed to biofilms and these numbers are noticeably increasing (Burke 2003). These infections are complicated to treat due to the physiological resistance of biofilms to antimicrobial agents and host immune response (Lebeaux et al. 2014; Ahmed et al. 2018; Khan et al. 2020; Singh et al. 2022; Miao et al. 2025; Liu et al. 2024).

A promising approach to mitigation of biofilms is thermal shock. Several studies have reported significant biofilm reduction via applying thermal shocks at elevated temperatures for *in vivo* medical purposes (Park et al. 2011; O'Toole et al. 2015; Ibelli et al. 2018; Ricker et al. 2018; Beckwith et al. 2020; Prasad et al. 2022; Shaikh et al. 2023; Zhang et al. 2024; Wang et al. 2025). O'Toole et al. investigated thermal mitigation of *Pseudomonas aeruginosa* biofilms *in vitro* at temperatures ranging from 50°C to 80°C for 1 to 30 min exposure times. They observed that bacterial population can be reduced by over six orders of magnitude depending on the time and temperature combination (O'Toole et al. 2015). It has also been reported that the efficacy of thermal shock can be enhanced when used in combination with antibiotics treatment. Antibiotics can substantially reduce the duration and/or temperature needed to eradicate biofilms by aiding in achieving a critical population decrease beyond which biofilms become non‐viable. In some studies, these combined treatments are reported to be immediate and synergistic, surpassing the combined efficacy of individual treatments (Ricker and Nuxoll 2017; Aljaafari et al. 2023), whereas, in some cases, this synergistic effect may manifest over a longer timescale, as Aljaafari et al. observed when applying combination treatments on *Staphylococcus epidermidis* (Aljaafari et al. 2024). Several other studies also reported these synergistic effects in their biofilm mitigation attempts (Beckwith et al. 2020; Alumutairi et al. 2020; Pijls et al. 2020; Aljaafari et al. 2021; Wang 2023a).

Thermal destruction of biofilms has been typically investigated *in vitro* by immersion of the biofilm in thermostatted media followed by destructive enumeration of the biofilm. Immersion enables prompt, precise, and uniform delivery of thermal shocks and facilitates *in vitro* studies on fundamental aspects of this approach. However, it is only possible in limited circumstances clinically, for example, within the lumen of a catheter. To be practical and applicable to a broader range of medical implants, thermal shock must be delivered via direct heating of the surface upon which the biofilm is growing; that is, its substrate. This is possible by applying thermoelectric or electro‐resistive heating for medical devices with a source of power, for example, pacemakers or neurostimulators, or using an alternating magnetic field to remotely heat an implant with a metallic or magnetically susceptible cladding (Coffel and Nuxoll 2015; Chopra et al. 2017; Bing et al. 2015).

Temperature variation has been known as an environmental stimulus that can affect biofilms' behavior, though only recently has its role in phenotypic changes in biofilms attracted attention. Nguyen et al. observed an increase in dispersion rate when *P. aeruginosa* biofilms were exposed to mild temperature elevations (Nguyen et al. 2015). Townsley and Yildiz reported a reverse trend when formation of *Vibrio cholerae* biofilms increased in response to downshift changes in temperature (Townsley and Yildiz 2015). Several studies associate this behavior with the intracellular content of a secondary messenger molecule, referred to as cyclic diguanosine monophosphate (c‐di‐GMP). Reduction of c‐di‐GMP results in biofilm dispersal while its production is a step towards building extracellular polymeric substance (Townsley and Yildiz 2015; Chua et al. 2014; Wang 2023b; Conner et al. 2017). These studies investigated the effects of small temperature elevations (≤ 10°C) on biofilm dispersion. While the biochemical reaction of biofilm bacteria to greater temperature upshifts remains unknown, such behavior may result in different biofilm destruction mechanisms under various thermal shock modalities. In immersion, the biofilm is exposed to a hostile temperature promptly and uniformly from all directions. This condition is expected to kill much of the planktonic bacteria dispersed from the biofilm into the hot media which cannot then be readily quantified, making the magnitude of dispersed bacteria unknown. On the other hand, surface heating is expected to be slower and less uniform due to the possibility of temperature gradients along the edges of the substrate and as heat is applied to the biofilm from one direction only, the surrounding environment is at a lower temperature wherein the dispersed bacteria may survive and repopulate the biofilm quickly after the shock ends. Given these differences, the efficacy of surface heating is expected to be different from immersion heating in elimination of bacterial biofilms.

The primary aim of this study was to investigate surface heating of *P. aeruginosa* biofilms cultured on a self‐heating substrate, that is, a thermoelectric device, to establish a baseline for the efficacy of biofilm destruction via this technique and determine differences in efficacy of surface heating *vs* immersion heating as a crucial step in development of thermal shock. Thermal shock was delivered via substrate heating to biofilms in static nutrient media at 60°C, 70°C, or 80°C for 1, 5, or 10 min followed by quantifying the bacterial population density in the biofilm and media. More fundamentally, baseline dispersion behavior of *P. aeruginosa* biofilms was characterized in both equilibrium and flow cell conditions. Biofilms were allowed to equilibrate with sequential 1‐liter aliquots of media to determine the time scale of equilibrium between dispersion and reattachment and the shift in this equilibrium as the dispersed bacteria are progressively removed. The dispersion rate was characterized independent of reattachment by mounting the biofilm in a flow cell (mean velocity 0.21 cm/s) and monitoring the effluent media.

## 2 Materials and Methods

### 2.1 Streak and Inoculum

*P. aeruginosa* PAO1 strain (15692, American Type Culture Collection, VA, USA) was thawed and streaked onto an agar dish (Difco Nutrient Agar, BD, MD, USA). The dish was inverted and incubated for 24 h at 37°C. Two colonies were collected with a sterile inoculum loop from the dish and suspended in 5 ml sterile tryptic soy broth (TSB) (Difco Tryptic Soy Broth, BD, MD, USA) prepared to the manufacturer's specification of 30 g/L. The inoculum was incubated for 24 h at 37°C to reach an average of ~10^9^ Colony Forming Units (CFU)/mL.

### 2.2 Biofilm Growth

To culture biofilms for thermal shock trials in static media, two 35 mm tall polypropylene cylinders with internal diameters of 27 and 38 mm were mounted on a thermoelectric device (TED), 40 × 40 × 3.2 mm (HP‐199‐1.4‐0.8, TE Technology, MI, USA) to form two concentric wells. To seal the inner cylinder to the surface, polystyrene resin (~280,000 MW, Sigma Aldrich, MO, USA) dissolved in toluene (Fisher Scientific, MA, USA) was used as a weak and inert adhesive. The outer well was sealed to the surface by silicone glue (General Electric, MA, USA). After curing the sealings for 24 h, a biofilm was cultured inside the inner well by diluting 100 μl of the inoculum into 1500 μL of 30 g/mL sterile TSB. The top of the well was covered with parafilm and incubated at 37°C on an orbital shaker table (VWR 1000, 15 mm orbit, PA, USA) set at 160 rpm. *P. aeruginosa* biofilms were cultured for 24 h. Control biofilms enumerated at various times established that the biofilms were then at a resting phase population density of 10^7.34 ± 0.25^ CFU/cm^2^. The inner cylinder and sealant were removed, leaving a 27‐mm‐wide flat biofilm which was entirely exposed to the uniform central region of the 40‐mm‐wide TED surface.

For trials studying equilibrium dispersion in static media, *P. aeruginosa* biofilms were grown onto microscope slides, 75 × 25 × 1 mm, fully frosted on one side (Leica Biosystems, IL, USA). Slides were placed in a 4‐well plate (Thermo Fisher Scientific, MA, USA) and 333 μL of the inoculum was added into each well containing 5 ml sterilized 30 g/L TSB. The plate was covered with parafilm and incubated at 37°C for 96 h on the orbital shaker table set at 160 rpm. The average population density of biofilms on microscope slides was 10^7.13 ± 0.58^ CFU/cm^2^.

For dispersion trials in the flow cell, the biofilm was cultured on a TED, 72.4 × 16.2 × 2.7 mm (XLT2427, II‐VI Marlow, TX, USA), by adding 666 μL of the inoculum into the wells containing the devices immersed in 10 mL 30 g/L TSB. The plate was covered with parafilm and incubated at 37°C on the shaker table for 96 h. The biofilms had an average population density of 10^7.02 ± 0.67^ CFU/cm^2^.

### 2.3 Enumeration Protocol

The population density of biofilms and media samples was quantified by direct enumeration. The biofilms were disrupted and resuspended in 5 or 10 mL fresh dilute TSB at room temperature by sonication at 45 kHz for 10 min (9.5 L VWR Symphony, PA, USA). Media samples did not require disruption. The samples were then serially diluted in 10‐fold ratios followed by spot plating 10 μL from each dilution on agar dishes. Each dilution of each sample was plated in duplicate. When the plated spots were absorbed by the agar (~15 min), the culture dishes were inverted and incubated at 37°C for 20–24 h. Within each dilution series, the dilution with the largest CFU count below 35 (to prevent overlap errors) was used to calculate the population density of the sample.

### 2.4 Thermal Shock Protocol in Static Media

The incubated media/supernatant in the well was pipetted out before decanting 5 mL sterilized diluted TSB (3 g/mL) into the well to remove the original planktonic bacteria in the growth area. After 1 min, the rinsing media was withdrawn by a pipette, and the inner well and the polystyrene adhesive were removed. Then, after adding 5 mL fresh dilute TSB to the remaining well and sealing the top with parafilm, the TED was placed in a water bath with its bottom surface in contact with 37°C water. The water bath served as an infinite heat source to ensure the TED supplied the necessary heat in the necessary time, regardless of the target temperature. Thermal shocks were applied at 37, 60, 70, or 80°C for 1, 5, or 30 min by driving power to the TED using a programmable DC power supply (1785‐B, BK‐Precision, CA, USA). Thermocouples (5SRTC‐TT‐K‐40‐36, Omega, CT, USA) connected to a data acquisition device (USB‐Temp, Measurement Computing, MA, USA) were placed on the TED's surface and in the surrounding media at the air/media interface to monitor the surface and bulk media temperatures. All devices were controlled via LabVIEW software (13.0.1, National Instruments, TX, USA) to minimize the ramping time and overshoot of the thermal shock and maintain the target temperature once it was achieved. In separate tests, the system was insulated, allowing the temperature of the surrounding media to increase more substantially during the thermal shock. The same shocks were performed in this insulated system to study the effect of media temperature on biofilm reduction. After the shock, the device was removed from the water bath and a sample from the media was enumerated immediately. Withdrawing the shocked media from the well and replacing it with 5 ml fresh dilute TSB, the enumeration protocol was undertaken to quantify the population density of the shocked biofilm.

### 2.5 Dispersion Equilibrium Trials in Static Media

The biofilm with its underlying microscope slide was immersed in 1000 mL dilute TSB at room temperature. Samples were taken after 1, 2, 3, 5, 10, 20, or 30 min from just below the liquid surface, directly above the slide and enumerated. In separate trials, the biofilm was resuspended and enumerated immediately, after every sampling time mentioned.

Additional trials were performed using three sequential dishes where after 5 min residence time in each dish, the slide was transferred directly to the next 1000 mL dish of fresh TSB (30 g/L) before being removed and enumerated, along with samples from the media of the three dishes.

### 2.6 Thermal Shock Protocol in the Flow Cell

For each flow cell trial, a biofilm grown on a 16‐mm‐wide TED was rinsed in 5 mL dilute TSB for 1 min to remove the original planktonic bacteria. Then, the biofilm with its underlying device was placed on the bottom surface and in the center of a custom‐built polyacrylic flow cell (150 × 25 × 25 mm). A stream of fresh dilute TSB at room temperature was pumped into the flow cell at a rate of 80 mL/min using a peristaltic pump (Masterflex L/S Brushless 7518‐10, Cole‐Parmer, IL, USA), giving a mean velocity of 0.21 cm/s). This flow rate was experimentally determined to avoid being so slow that the media temperature might rise enough to kill any dispersed bacteria, and avoid being so large it might mechanically debride the biofilm (monitored visually) or dilute the dispersed bacteria beyond the lower limit of the enumeration assay. The flow cell effluent was sampled and enumerated at 0.5, 1, 5, 10, 20, and 30 min after a steady stream was established at the outlet. After 40 min, during which the effluent bacteria population appeared to decrease to a fairly constant dispersion baseline, thermal shock at 70°C for 5 min was delivered using the setup explained earlier. The flow cell effluent was again frequently sampled and enumerated during and after the thermal shock. Five min after the thermal shock, the biofilm on the TED was resuspended and enumerated.

### 2.7 Statistical Analysis

Statistical analysis of the enumeration results was performed in Minitab 20 (Minitab Statistical Software, State College, USA). One‐way analysis of variance (ANOVA) with a 95% confidence interval was used to compare the means.

## 3 Results

### 3.1 Surface and Media Temperature for Surface Thermal Shocks

**Figure 1:**
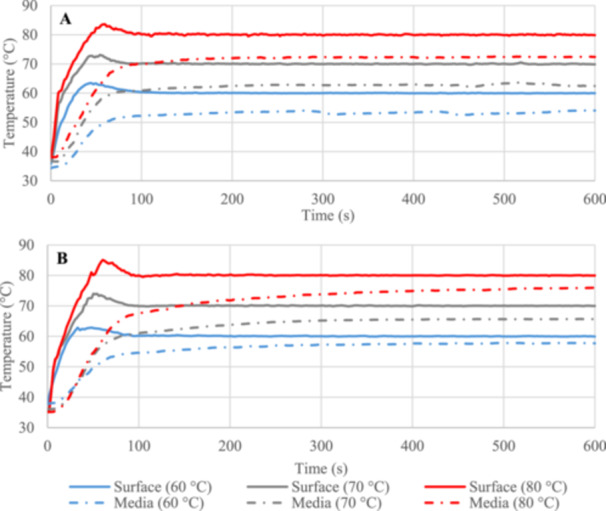
Temperature changes on the TED's surface and in the media contained in the uninsulated (A) and insulated (B) well for 60°C, 70°C, and 80°C thermal shocks applied for 600 s.

Targeting 60°C surface temperature in the uninsulated trials, the TED reached the setpoint in about 30 s from its initial temperature (~37°C). Reaching 70°C and 80°C setpoints was slower, requiring 36 and 45 s, respectively. Media temperature measured at the air‐media interface was always lower than the surface temperature at all setpoint conditions over the course of the 10 min heating. For 60°C thermal shock the smallest gap between media and surface temperature was roughly 6°C with media reaching a maximum of 54°C. For 70°C thermal shock the media reached a steady maximum of approximately 63°C and when the TED surface was controlled at 80°C the media plateaued at 72°C (see Figure 1A). Each case indicates that the surrounding ~20°C air already provided about 85% of the resistance to heat transport from the TED surface to the surrounding air.

To demonstrate the effect of higher media temperatures, the trials were repeated after insulating the system. As shown in Figure 1B, under new conditions the media temperature plateaued at 57°C, 66°C, and 76°C for 60°C, 70°C, and 80°C surface temperatures, respectively, indicating that the insulation doubled the resistance of the surroundings, dropping the temperature gradient within the media in half.

**Figure 2:**
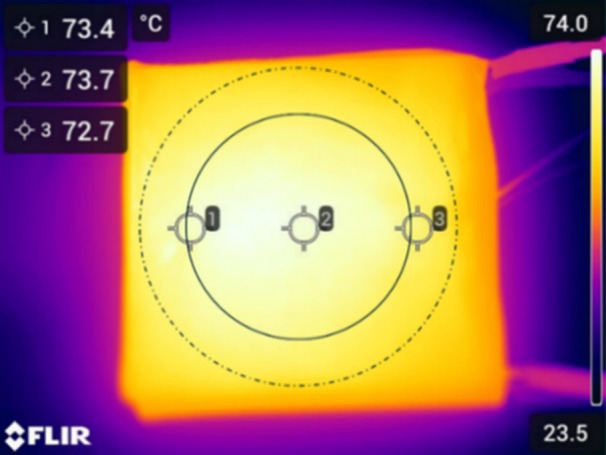
Thermal map of the TED. The solid circle marks the area where the biofilm was grown, and the dashed circle depicts the perimeter of the outer well on the device.

Another complication of surface heating is providing a uniform temperature across the entire biofilm, particularly at the edges of the surface which typically have greater exposure to the surrounding heat sink. In these trials, the biofilm was limited to the middle 27 mm of the surface, which as shown in Figure 2 had less than 1°C variation across it.

### 3.2 Surface Heating of Biofilms in Static Media (Uninsulated System)

**Figure 3:**
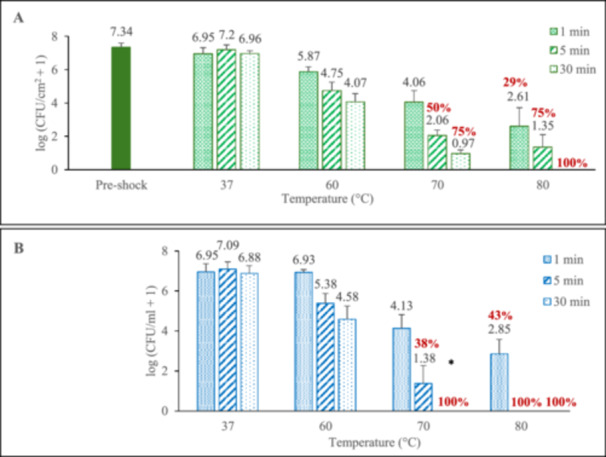
Thermal susceptibility of *P. aeruginosa* biofilms (A) and surrounding media (B) following surface thermal shock. Red values show percentage of shocked biofilms with no detected CFUs immediately after their thermal shock. Error bars represent standard deviation for biofilms with detected CFUs. At least eight biofilms were tested at each elevated temperature/time combination, with four biofilms tested at each 37°C exposure time.

Biofilms were ‘shocked’ at their incubation temperature of 37°C to confirm that the shock protocol itself did not significantly alter the biofilm population density. As shown in Figure 3A, pre‐shock (control) biofilms had an average population density of 10^7.34 ± 0.22^ log(CFU/cm^2^) and the population of shocked biofilms at 37°C was not statistically different from the control group, regardless of exposure time (*p* = 0.494). Figure 3A also demonstrates that thermal shock does significantly reduce biofilm population density. Delivering shocks at 60°C, 1 min exposure resulted in a 1.5 log reduction (i.e., reduced by a factor of 10^1.5^, or 32). Increasing the exposure to 5 min appeared to reduce the bacterial population by another order of magnitude, but the additional reduction from extending the exposure by another 25 min was less than one log. At 70°C, however, just 1 min was enough to decrease the biofilm population density by 3.3 orders of magnitude. Thermal shocks at 70°C for 5 min decreased the bacterial population even more substantially, often below 10^0.82^ CFU/cm^2^, the lower limit of the enumeration assay. After 30 min exposure to 70°C, only a quarter of the biofilms survived the harsh thermal shock having an average population density of 10^0.97 ± 0.21^ CFU/cm^2^. Moving on to 80°C thermal shocks, 1 min exposure reduced the population density by roughly five orders of magnitude. Exposure to 80°C for 5 min killed 75% of the biofilms and reduced the population density of the surviving ones by over 6 log (i.e., by over 99.9999%). Furthering the shock to 30 min, no CFUs were detected. Bacterial population in the corresponding media tracked these values closely, as shown in Figure 3B.

### 3.3 Surface Heating of Biofilms in Static Media (Insulated System)

**Figure 4:**
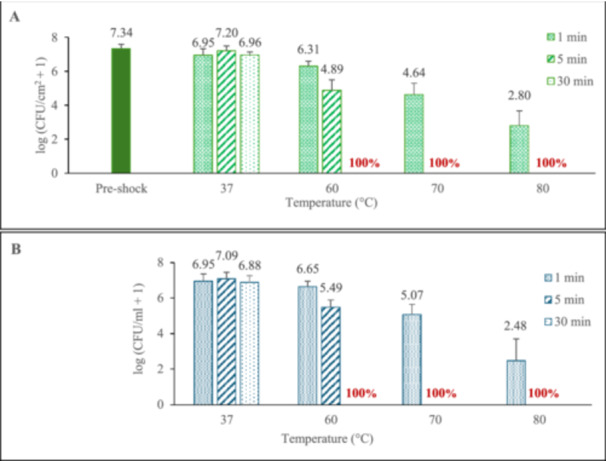
Thermal susceptibility of *P. aeruginosa* biofilms in the insulated wells (A), and population density of the bacteria in the media, immediately after the thermal shock (B). Red values show percentage of total samples with no detected CFUs after their thermal shock. Error bars are standard deviation of samples with detected CFUs. At least eight biofilms were tested at each elevated temperature/time combination, with four biofilms tested at each 37°C exposure time.

Repeating these trials in the insulated system where the media temperature more closely matched the biofilm temperature, significantly greater biofilm population reductions were observed (Figure 4). Thirty min thermal shocks at any elevated temperature eliminated all biofilms, as did 5 min thermal shocks at 70 or 80 C. Even 1 min at 80°C (45 s of which was ramp‐up) dropped the biofilm population by over 4 orders of magnitude. Notably, the population density of the surrounding media again closely tracked the population density of its corresponding biofilm.

### 3.4 Dispersion Equilibrium in Static Media

In the equilibrium trials, when the biofilm was immersed in the dish with fresh dilute TSB, the media population density reached 10^5.00 ± 0.82^ CFU/mL in just 1 min and remained statistically constant regardless of retention time in the media (Four different biofilms were tested at each sampling time; *p* = 0.686).

In trials involving sequential transfer of the biofilm to the second and third dishes, following 5 min of retention in each, the equilibrium ratio between the media and biofilm was initially measured at 0.84 log(CFU/mL)/log(CFU/cm^2^) in the first dish, almost consistent with values observed in the thermal shock trials (Table 1). However, this ratio decreased to 0.59 log(CFU/ml)/log(CFU/cm^2^) in the third dish indicating that some bacteria adhere to the surface more strongly than others and cannot be simply rinsed away from the biofilm. This behavior aligns with the proliferation of biofilms observed in both natural environments and man‐made systems, particularly in instances where they form and propagate within a flowing bulk fluid (Chun et al. 2022; Papadatou et al. 2021).

**Table 1:** Ratio of *P. aeruginosa* population density between the media and biofilm under different thermal shock conditions. Ratios are provided for population densities greater than the quantification limit.

Thermal shock	Media/Biofilm (Uninsulated) log(CFU/ml)/log(CFU/cm^2^)	Media/Biofilm (Insulate) log(CFU/ml)/log(CFU/cm^2^)
37°C for 1 min	1.00	1.00
37°C for 5 min	0.98	0.98
37°C for 30 min	0.99	0.99
60°C for 1 min	1.18	1.05
60°C for 5 min	1.13	1.12
60°C for 30 min	1.13	—
70°C for 1 min	1.02	1.09
70°C for 5 min	—	—
70°C for 30 min	—	—
80°C for 1 min	1.09	0.89
80°C for 5 min	—	—
80°C for 30 min	—	—

### 3.5 Dispersion Equilibrium and Thermal Shock Effect in Flow Cell

**Figure 5:**
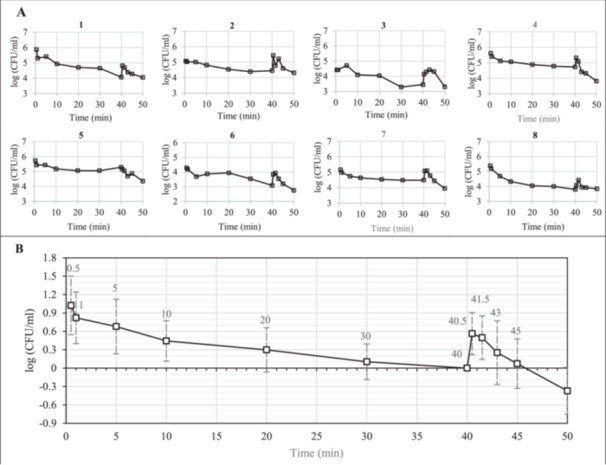
Trend of dispersion from eight different biofilms (A) exposed to 80 ml/min stream of dilute TSB in a flow cell where thermal shock at 70°C for 5 min was applied at the 40th min. The average trend of dispersion from the eight biofilms (B). Datapoints are normalized by the population density at the 40th min. Error bars show standard deviations of averages.

Figure 5A depicts the population density of effluent from eight flow cell trials, each starting with ambient temperature media and implementing a 70°C, 5 min thermal shock starting at the 40th minute. Such trials contain significant noise, but consistently show an initially steep population decline followed by a more gradual decrease until the 40th minute, at which point the bacterial population in the effluent spikes by an order of magnitude before decreasing at a higher rate than before the shock. These trials, normalized by their population density immediately prior to the thermal shock, are compiled in Figure 5B.

## 4 Discussion

### 4.1 Complications of Surface Versus Immersion Heating

One key advantage of immersion heating for investigating thermal shock is the immediate, precise, uniform heating provided by a large sink of thermostatted media heating a biofilm less than 100 μm thick and a substrate perhaps only a mm thick. The time required to raise the biofilm from its initial ambient temperature to its target temperature (“ramp time”) is much less than a second, and the variation in temperature across the biofilm during this time and the subsequent “hold time” of the shock is much less than 1°C. The uniformity and time lag of returning the biofilm to ambient temperature at the end of the shock is similar.

For clinical implementation, however, the thermal shock must be delivered precisely at the device surface where the biofilm is growing, while leaving the surrounding tissue as cool as possible. If an onboard power supply and wireless telemetry is available with the device, TED's provide the particular advantage of pumping heat from the underlying device, chilling it as they raise the temperature of the biofilm. At the end of the shock, the cool underlying device can then provide a heat sink for cooling the outer surface back to physiological temperature without requiring all of the heat to dissipate through the surrounding tissue, potentially causing much more damage. The thermal profiles in Figure 1 demonstrate the ramp‐up time required for such heating, which must be accounted for in the target exposure time, particularly for the short, high‐temperature exposure times which previous studies have indicated provide more consistent deactivation and lower tissue damage (Ricker et al. 2018). In the 80°C, 1 min trials, the ramp‐up constituted 75% of the total exposure time. The 30 min trials, while having much less of the exposure time devoted to ramp‐up, may also slightly understate the effect of thermal shock as they allow more time for bacterial growth once the system is below its resting‐phase population density. If the 37°C growth rate could be maintained during the 30 min shocks, it could increase the remaining population densities by up to 0.3 log(CFU/cm^2^). Uniform temperature across the entire biofilm is also critical, and achieved in these trials as shown in Figure 2 by constricting the biofilm to the center 27 mm of the TED. More careful design of device edges would be required for clinical implementation.

### 4.2 Higher Media Temperatures Maintained Same Bacterial Equilibrium but Increased Bacterial Death

More importantly, Figure 1A shows that the surrounding media provided about 15% of the temperature drop between the biofilm and the surrounding ~20°C air, and therefore about 15% of the thermal resistance. By insulating the system, the temperature difference between the biofilm and the surrounding media decreased by half, as shown in Figure 1B, meaning the insulation doubled the overall thermal resistance.

**Figure 6:**
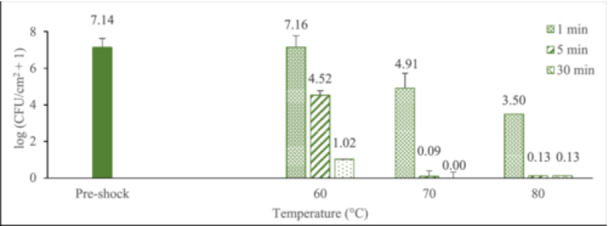
Thermal susceptibility of *P. aeruginosa* biofilms delivered with immersion thermal shock. Data from (Al‐Jaafari 2022).

Comparing the biofilm results of the uninsulated system (Figure 3A) with the results reported by Aljaafari et al (Al‐Jaafari 2022). on thermal susceptibility of *P. aeruginosa* biofilms exposed to immersion heating (Figure 6), it is evident that surface shock does not reduce biofilm population as well as immersion shock. Surface heating was particularly less effective when shock exposure was longer, that is, 5 or 30 min.

One hypothesis for this lower efficacy is that the bacteria rapidly interchange between the hot surface and the cooler surrounding media, avoiding some thermal exposure. This implies a rapid equilibrium between the surface and the media. Comparing the population density in the biofilm to its surrounding media immediately following the thermal shock, this apparent equilibrium between the biofilm and the media is evidenced by a constant ratio between the population density on the surface and within the media across all trials, including controls, as shown in Table 1. The speed for reaching such equilibrium is demonstrated by the equilibrium trial results, where the media reached a plateau concentration within 1 min, further showing that this interchange between surface and media occurs fast enough to maintain an equilibrium even in a 1 min thermal shock.

This hypothesis was tested by insulating the system so that the media is no longer significantly cooler than the biofilm for most of the trial, without changing any other parameter in the system. The biofilms yielded significantly lower populations afterwards, as shown in Figure 5, and media populations were still tracking the biofilm populations (also shown in Table 1). This strongly suggests the mechanism by which surface heating is handicapped in eliminating biofilms *in situ* which must be considered in developing self‐sterilizing implant surfaces.

### 4.3 Thermal Shock Triggers Increased Dispersion Rate in Flow Cell Biofilms

Multi‐dish equilibrium trials demonstrated that a subpopulation of the biofilm was not as prone to dispersion and could not be simply rinsed away by continuously providing fresh media. This behavior was demonstrated in a different way in the flow cell trials. If the ratio of bacteria in the media versus biofilm stayed constant as the media was continually flushed from the system, the population density in the effluent media (and the biofilm) would decrease logarithmically with time, giving a straight line with a downward slope in each panel of Figure 5. When a thermal shock was applied in the flow cell, the dispersion rate spiked significantly and immediately (see Figure 5B). Notably, when accounting for the initial sampling time after the thermal shock (30 s) and the lag time of the flow cell to receive a signal at the outlet (approximately 20 s), it becomes evident that the biofilm's response rate to the shock was less than 10 s. This observation aligns with findings reported by Nguyen et al. where they observed that exposing *P. aeruginosa* biofilms to slight temperature increases led to an enhanced dispersion rate. Specifically, they noted the strongest response when the temperature was raised from 37°C to 45°C, resulting in over a threefold increase in dispersed bacteria compared to the baseline dispersion levels (Nguyen et al. 2015). The increased dispersion resulting from thermal exposure may contribute to the perceived synergy observed when combining antibiotics with thermal shock. This synergy arises from the fact that thermal shock not only eradicates bacteria (both in biofilm and planktonic form), but also alters the fraction of bacteria susceptible to antibiotics, the planktonic fraction, by inducing dispersion.

Bacterial dispersion is a fundamental component of all biofilms and their thermal mitigation has been studied for other common pathogenic bacterial such as *S. aureus* and *S. epidermidis (*Aljaafari et al. 2024, 2023). The rapid interchange between planktonic and biofilm bacteria and the increased dispersal rate in response to aggressive thermal shock have not yet been systematically studied for those species. However, the synergism observed between thermal shock and antibiotics in mitigating those biofilms supports the hypothesis that their increased dispersion to a more antibiotic‐susceptible phenotype in response to aggressive thermal shock is similar to *P. aeruginosa's*. This also underscores a practical consequence of this dispersion *in vivo*. The bloodstream may quickly carry dispersed bacteria permanently beyond reach of the thermal shock, and increasing the dispersed population could have dire health consequences for the patient. Antibiotic synergism, or at least orthogonality, indicates that these dispersed bacteria, unlike their biofilm phenotype, are still susceptible to antibiotics. Increasing systemic antibiotic and immune system activity may be a critical corequisite to localized thermal shock of biofilms along with inflammation to remediate any thermal damage to the tissue immediately adjacent to the device surface.

Instead, at early times (higher population densities) the decrease is more rapid (steeper slope) than at later times (lower population densities), again showing that the last 10% of the bacteria are less inclined to disperse than the first 90%. In literature, these variations in biofilm dispersion rate are linked to bacterial quorum sensing which is reported to depend on high cell density, and therefore high concentrations of signal molecules (Davies et al. 1998; Heydorn et al. 2002; Liu et al. 2025; Hernández‐Valle et al. 2024). In open systems with continuous renewal of bulk media, these diffusible signal molecules may be washed away by the sink of fresh media along with the dispersed bacteria. Consequently, the signal concentration within an open system is anticipated to be influenced by the production rate, the rate of diffusion through the biofilm, and the hydrodynamic conditions of the bulk liquid (Purevdorj et al. 2002). This flow cell system might be expected to enhance dispersion over time as both the signal molecules and the bacteria that produce them decrease in concentration, progressively decreasing the quorum cues for biofilm formation, but rather the opposite is observed.

## 5 Conclusions

Biofilm infection on medical implants is a major problem which has not been solved by conventional approaches despite decades of research and refinement. New approaches are needed, such as thermal mitigation of the biofilm *in situ*. Thermal shock has been demonstrated to effectively eliminate bacterial biofilms at a variety of time temperature combinations and can be augmented by other strategies such as antibiotics. Delivery of this thermal shock by the substrate on which the biofilm grows may be complicated, however, by the bacteria's ability to disperse away from the biofilm into cooler surroundings, then later rejoin the biofilm, partially escaping the thermal shock. This study showed that upon immersion in a fixed amount of media, bacteria from the biofilm dispersed to a constant population density within seconds. Thermally shocking a biofilm from its substrate, the population density of bacteria in both the biofilm and the surrounding media decreased comparably across a wide range of thermal exposures, despite the fact that the media was at a lower temperature than the substrate. When the system was insulated to make the media temperature more closely match the substrate, the bacterial population density decrease was enhanced not just in the media, but in the biofilm as well, though the biofilm experienced the same thermal shocks as before. This demonstrates that the bacteria are rapidly exchanging between the biofilm and the surrounding media, so enhancing the thermal shock in one phase enhances the bacterial death in both. Viewed conversely, minimizing the thermal shock in the surroundings will reduce bacterial death in the biofilm. Hence thermally shocking the biofilm via its substrate while minimizing the shock to the surrounding tissue may reduce the thermal shock's efficacy. Flow cell results indicating that thermal shock triggers an increased dispersion rate enhance this concern. A better understanding of biofilm dispersion, particularly into surrounding tissue, is needed accurately determine the thermal shock needed to reliably eliminate the biofilm while minimizing damage to the surrounding tissue.

## Author Contributions

**Parham Parnian:** conceptualization, methodology, formal analysis, investigation, visualization, writing – original draft, writing – review and editing. **Paraskevi K. Zoga:** investigation. **Haydar A. S. Aljaafari:** methodology. **Hannah Chicchelly:** investigation. **Michael Toops:** investigation. **Eric Nuxoll:** conceptualization, methodology, writing – original draft, writing – review and editing, resources, supervision, funding acquisition, project administration.

## Ethics Statement

The authors have nothing to report.

## Conflicts of Interest

Eric Nuxoll reports financial support was provided by American Heart Association. Parham Parnian reports administrative support was provided by National Institute of Health. Eric Nuxoll has a patent for a thermal shock coating through the University of Iowa Research Foundation. The remaining authors declare no conflicts of interest.

## Data Availability

The data that support the findings of this study are openly available in Harvard DataVerse at https://dataverse.harvard.edu/dataset.xhtml?persistentId=doi:10.7910/DVN/RINXWE.
